# The prognostic relevance of interactions between venous invasion, lymph node involvement and distant metastases in renal cell carcinoma after radical nephrectomy

**DOI:** 10.1186/1471-2490-8-19

**Published:** 2008-12-19

**Authors:** Dragomir P Zubac, Leif Bostad, Tomas Seidal, Tore Wentzel-Larsen, Svein A Haukaas

**Affiliations:** 1 Department of Surgical Sciences, University of Bergen, Norway; 2 Department of Pathology, Haukeland University Hospital, Bergen, Norway; 3 Department of Pathology, Central Hospital, Karlstad, Sweden; 4 Center for Clinical Research, Haukeland University Hospital, Bergen, Norway; 5 Department of Surgery, Section of Urology, Haukeland University Hospital, Bergen, Norway

## Abstract

**Background:**

To investigate a possible prognostic significance of interactions between lymph node invasion (LNI), synchronous distant metastases (SDM), and venous invasion (VI) adjusted for mode of detection, Eastern Cooperative Oncology Group performance status (ECOG PS), erythrocyte sedimentation rate (ESR) and tumour size (TS) in 196 patients with renal cell carcinoma treated with radical nephrectomy.

**Methods:**

Median follow-up was 5.5 years (mean 6.9 years; range 0.01–19.4). The mode of detection, ECOG PS, ESR and TS were obtained from the patients' records. Vena cava invasion and distant metastases were detected by preoperative imaging. The surgical specimens were examined for pathological stage, LNI and VI.

**Results:**

The univariate analyses showed significant impact of VI, LNI, SDM, ESR and TS (p < 0.001), as well as mode of detection (p = 0.003) and ECOG PS (p = 0.002) on cancer specific survival. In multivariate analyses LNI was significantly associated with survival only in patients without SDM or VI (p < 0.001) with a hazard ratio of 9.0. LNI lost its prognostic significance when SDM or VI was present.

**Conclusion:**

Our findings underline the prognostic importance of the status of the lymph nodes. LNI, SDM, ESR, and VI were independently associated with cancer specific survival after radical nephrectomy. LNI provided the strongest prognostic information for patients without SDM or VI whereas SDM and VI had strongest impact on survival when there was no nodal involvement.

## Background

Renal cell carcinoma (RCC) is the third most common genitourinary malignancy and accounts for 3% of cancer in adults. About 25% of patients with RCC present with metastatic disease; either lymph node infiltration, simultaneous distant metastases or both [1].

The wide application of imaging modalities has increased the incidental detection of RCC and changed its natural history over the last two decades. However, it has been difficult to find reliable prognostic factors particularly for locally advanced and metastatic RCC. The prognostic value of mode of presentation and Eastern Cooperative Oncology Group performance status (ECOG PS) in RCC have been controversial in earlier studies [2,3]. Even though ECOG PS and symptoms at presentation were of independent prognostic significance, the combination of those two variables in prognostic models did not improve the capability to predict RCC specific mortality [3]. The contribution of tumour size (TS), erythrocyte sedimentation rate (ESR) and venous invasion (VI) in prediction of prognosis have been a matter of debate in several studies [2,4-7]. According to recent studies the presence but not the extent of venous invasion was independently correlated with the cancer-specific survival [8,9].

One of the most important prognostic factors in RCC is lymph node invasion (LNI). However, the prognostic discrimination between pN1 and pN2 categories in the 2002 TNM system has been questioned. A recent study concludes that the percentage of positive nodes and a threshold number of four rather than one positive lymph node correlated significantly with clinical outcome [10]. The appropriateness of the pNx/pN0 grouping and prognostic relevance in a multivariate setting has also been discussed [11].

To our knowledge the possible impact on cancer specific survival (CSS) of interactions between LNI, synchronous distant metastases (SDM) and VI have not yet been fully studied. We hypothesized that putatively significant interactions between these variables could contribute in prediction of outcome for patients with RCC after radical nephrectomy.

To test this hypothesis we examined the relationship between cancer specific survival and LNI, SDM, and VI, including possible interactions between these variables, with adjustment for mode of detection, Eastern Cooperative Oncology Group performance status, erythrocyte sedimentation rate and continuously coded tumour size.

## Methods

### Patients

The patient material in this retrospective and population-based series is described in detail elsewhere [12]. Briefly, a total of 196 consecutive patients who underwent radical nephrectomy for renal cell carcinoma during the years 1985 to 1994 were included in the study. Tumour staging was done according to the 2002 TNM classification system using the American Joint Committee on Cancer (AJCC) stage grouping [13].

All patients were given a thorough clinical examination preoperatively, including imaging techniques. Bone scans were performed in those with symptoms and signs of skeletal involvement. When a thrombus of the inferior vena cava was suspected, both ultrasound and an inferior cavogram were done in order to better define the thrombus level.

Approval to use the biological material for research purposes was granted in 2004 by the local authority at Karlstad Central Hospital in Sweden according to Swedish regulations. In Norway the appropriate Norwegian authority, Norwegian Social Science Data Services, recognized this approval. The study was carried out in accordance with the standards of World Medical Association Declaration of Helsinki as revised in 2008.

### Treatment

Standard radical nephrectomy was done in all patients. A thrombus of the vena cava was found in 15 patients and treated with cavotomy and thrombectomy. The clinical lymph node staging was performed based on preoperative CT images of the abdomen and/or the peroperative findings. In cases with enlarged or palpable lymph nodes between the aorta and vena cava or other sites, additional lymph node dissection was done. Extensive radical retroperitoneal lymph node dissection was not performed. Only 14 of the surgical specimens had a sufficient number of negative nodes (eight) to be classified as pN0 category. Accordingly 161 of the patients were pNx (clinically N0).

### Methods

Clinical information regarding age, sex, Eastern Cooperative Oncology Group performance status [14], erythrocyte sedimentation rate obtained preoperatively according to the Westergren method (reference interval: <28 mm/h) [15,16], metastases and final disease status were extracted from the patient's clinical records.

According to the mode of detection patients were classified in two groups: incidental and symptomatic. Tumours diagnosed during abdominal imaging studies for signs and symptoms unrelated to RCC were classified as incidental.

The clinical records and pathology reports were reviewed to determine stage, size and type of the primary tumour. Continuously coded tumour size (CCTS) was used in all analyses and given in cm by measuring the greatest diameter. The nephrectomy specimens were examined histopathologically in a uniform manner. Histological examination included searching for invasion of the tumour into perinephric or peripelvic fat, any possible involvement of the adrenals or spread beyond Gerota's fascia. Careful examination of the regional lymph nodes was done. One urologic pathologist (LB) performed the histological subtyping of the RCC according to the Heidelberg classification guidelines [17]. Venous invasion was registered as no venous invasion (pV0), renal vein invasion (RVI) (pV1) or vena cava invasion (VCI) (pV2) and dichotomised (pV0 vs. pV1+pV2) in all except descriptive analyses, for compatibility with most previous reports. RVI was diagnosed when there was invasion by tumour of major extra renal veins found microscopically in transverse slices of the vein. In patients with VCI, the tumour thrombus did not adhere to the intima of the vena cava.

### Follow-up

The postoperative clinical follow-up was done at each hospital according to established protocols, including clinical and radiological examination at regular intervals for a median of 3 years (range 0.1–11.5). Information of local recurrence, metastases and final status were extracted from hospital files or from information obtained by contacting the local general practitioners. The cause of death was determined from clinical records and death certificates. Deaths from other causes than RCC were censored. We obtained information of surviving patients by searching The Swedish Updated Population Register and by contacting the local general practitioners (GPs). The clinical status was complete up to 30 April 2004 when the study was ended. Thus, the patients could be assigned a date of death or identified as being alive with or without diagnosed recurrent disease. Median and mean follow up period for the whole population studied was 5.5 years and 6.9 years (range 0.01–19.4), respectively. Median follow up was 13.9 years for patients alive at the end of the study.

### Statistical procedures

Preliminary analyses included descriptive statistics and assessment of associations of LNI, SDM and VI with TS, presenting symptoms and ECOG PS, by cross tabulations, with exact χ^2^-, linear by linear association or Mann-Whitney tests. The relationships of CSS to LNI, SDM and VI were investigated by multiple Cox regression analysis for CSS, including all two-way interactions and adjusting for ESR, CCTS (continuously coded), presenting symptoms and ECOG PS. All statistical analyses were accomplished in SPSS 14.0 (SPSS Inc., Chicago, USA). All tests were two-sided with a significance level at 0.05.

## Results

### Predictive factors

The clinicopathological characteristics of the 196 patients in the study are detailed in Table 1. In the symptomatic group the leading symptom was haematuria in 64, weight loss associated with asthenia 14, or flank pain 42. Abnormal blood tests like elevated erythrocyte sedimentation rate, anemia or elevated haemoglobin were the main causes for detection of RCC in 25, 8 and 2 patients, respectively. At presentation 45 patients harboured distant metastases and about three quarters had metastases in multiple sites; the most frequent sites were lungs (24 patients), the skeleton (10), the liver (5), the brain or the spine (4) and other sites (14).

**Table 1:** Clinical and pathologic features in 196 patients with renal cell carcinoma.

Features	Clear cell carcinoma	Papillary carcinoma	All subtypes^a^
No. of cases	172 (87.8)	19 (9.7)	196 (100)

Mean age (years)	66.6	62.4	66.4

Gender			
n Male/Female	95/77	13/6	110/86 (56.1)

Pathologic tumour size (cm)			
Median (range)	7.0 (1.5 – 26.0)	8 (3.0–20.0)	7 (1.5 – 26.0)

Mode of presentation			
Symptomatic/Incidental	144/28	17/2	165/31 (84.2)

ECOG performance status:			
0	109 (63.4)	12 (63.2)	125 (63.8)
1	52 (30,2)	6 (31.6)	58 (29.6)
2	11 (6.4)	1 (5.3)	13 (6.6)

ESR			
< 28 mm	88 (51.2)	10 (52.5)	103 (52.6)
≥ 28 mm	84 (48.8)	9 (47.3)	95 (48.5)

RCC Stage groups (TNM, 2002)			
I	52 (30.2)	7 (36,8)	62 (31.6)
II	24 (14.0)	5 (26,3)	29 (14.8)
III	48 (27.9)	3 (15.8)	53 (27.0)
IV	48 (27.9)	4 (21.1)	52 (27.0)

Primary pathological tumour stage (pT, 2002)			
pT1a	18 (10.5)	3 (15,8)	22 (11.2)
pT1b	39 (22.7)	5 (26.3)	46 (23.5)
pT2	28 (16.3)	6 (31.6)	34 (17.3)
pT3a	28 (16.3)	0 (0)	29 (14.8)
pT3b	48 (28.0)	5 (26.3)	54 (27.6)
pT3c	0 (0)	0 (0)	0 (0)
pT4	11 (6.4)	0 (0)	11 (5.6)

Pathologic stage of lymph nodes (N, 2002)			
pN x	141 (82.0)	15 (79.0)	161 (82.1)
pN 0	14 (8.1)	0 (0)	14 (7.1)
pN 1	10 (5.8)	2 (11.0)	12 (6.1)
pN 2	7 (4.1)	2 (11.0)	9 (4.6)

SDM (M, 2002)			
M 0	131 (76.1)	15 (79.0)	151 (77.0)
M + (single)	9 (5.2)	1 (5.3)	10 (5.1)
M + (multiple)	32 (18.6)	3 (15.8)	35 (17.9)

Venous invasion (VI = pV1+pV2)			
pV 0	122 (70.9)	14 (73.7)	140 (71.4)
pV 1 (renal vein)	36 (20.9)	4 (21.1)	41 (20.9)
pV 2 (vena cava)	14 (8.1)	1 (5.3)	15 (7.7)

Unless stated otherwise, values shown represent numbers of patients, with percentages in parentheses.

^a^Including chromophobic carcinoma (n = 5)

Lymph node invasion was diagnosed in 21 of 196 patients of whom 16 had SDM. In ten patients with LNI there was also venous invasion. Of 15 patients with vena cava invasion seven patients had SDM and six had regional local lymph node metastases. Venous invasion carried a significantly higher risk for having SDM (p = 0.003) as well as LNI (p = 0.014). Such patients also had a significantly higher rate of lung metastases as 16 patients with VI had lung metastases as compared to 9 patients without VI (p < 0.001). Additionally, venous invasion was significantly associated with tumour size (p < 0.001).

### Univariate analyses

As depicted in Table 2 factors such as age and gender were not associated with CSS. The mode of presentation and performance status was significant prognostic variables (p = 0.003 and p = 0.002, respectively). High ESR and large TS were inversely related to survival (p < 0.001). LNI, SDM and VI were also significantly related to CSS (p < 0.001) with hazard ratios of 5.4, 6.6 and 2.3, respectively.

**Table 2:** Univariate analysis of cancer specific survival related to clinicopathological variables in 196 patients operated with radical nephrectomy for renal cell carcinoma.

UNIVARIATE ANALYSIS	Cancer specific survival		
	HR	95% CI	p-value
Primary pathological tumour stage (pT, 2002)			
pT1a ref.			
pT1b	5.5	0,7 – 42.7	0.102
pT2	11.5	1,5 – 87.6	0.018
pT3a	16.3	2.2 – 123.5	0.007
pT3b	21.5	2.9 – 157.1	0.003
pT4	63.5	8.1 – 498.2	<0.001

Gender male ref.	0.74	0.48 – 1.14	0.170

Age (continuous), per 5 years	1.01	0.91 – 1.11	0.908

Continuously coded tumour size (CCTS), per 10 cm	2.98	1.87 – 4.75	<0.001

Mode of detection (Incidental vs. Symptomatic)	3.89	1.58 – 9.59	0.003

ECOG PS (≥ 1 vs. 0)	1.94	1.26 – 2.98	0.002

ESR (≥ 28 mm vs. < 28 mm)	3.36	2.11 – 5.35	<0.001

LNI (pN 1/2 vs. pN x/0)	5.38	3.17 – 9.10	<0.001

SDM (M + vs. M 0)	6.56	4.17 – 10.33	<0.001

VI (pV 1/2 vs. pV 0)	2.33	1.51 – 3.60	<0. 001

### Multivariate analyses

In multivariate Cox regression analysis, only LNI, SDM, VI and ESR remained independent prognostic factors (Table 3). Lymph node metastases had a significant impact on CSS only for patients in whom neither distant metastases nor venous invasion were present. In patients without lymph node metastases, we were able to define prognostic subgroups of patients based on multivariate analysis by including different combinations of SMD and VI.

**Table 3:** Multivariate survival analysis of interactions between clinicopathological variables and cancer specific survival in 196 patients operated with radical nephrectomy for renal cell carcinoma.

MULTIVARIATE	Cancer specific survival		
ANALYSES	HR	95% CI	p-value
Lymph Node Involvement present			
VI present, SDM present	0.55	0.23 – 1.30	0.173
VI present, SDM absent	2.78	0.80 – 9.64	0.108
VI absent, SDM present	1.77	0.74 – 4.21	0.197
VI absent, SDM absent	8.96	3.27 – 24.56	<0.001

Venous invasion present			
LNI present	0.63	0.25 – 1.64	0.349
LNI absent	2.05	1.22 – 3.43	0.006

Synchronous distant metastases present			
LNI present	0.93	0.32 – 2.74	0.894
LNI absent	4.71	2.68 – 8.27	<0.001

CCTS (Tumour size per 10 cm)	1.49	0.75 – 2.95	0.256

ESR (≥ 28 mm vs. <28 mm ref.)	1.94	1.15 – 3.26	0.012

ECOG PS (≥ 1 vs. 0)	1.47	0.93 – 2.33	0.099

Mode of detection (Symptomatic vs. Incidental)	0.66	0.25 – 1.71	0.392

**Figure 1:**
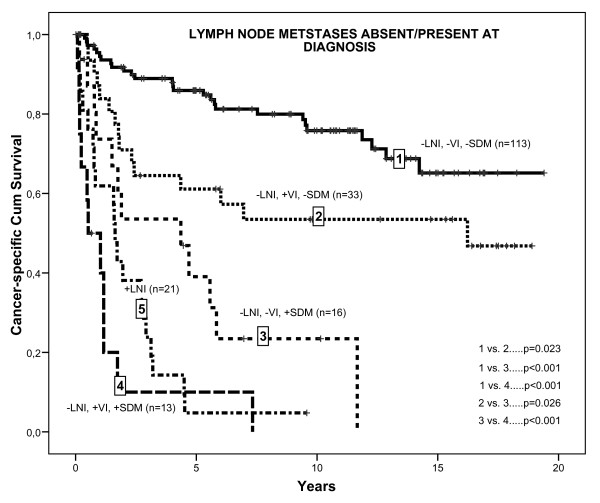
**Kaplan-Meier analyses of cancer specific survival in 175 lymph node negative renal cell carcinoma patients by different combinations of synchronous distant metastases (SDM) and venous invasion (VI) and in 21 patients with lymph node positive renal cell carcinoma**.

A Kaplan-Meyer analysis of CSS in node positive, as well as four combinations of SDM and VI in node negative patients is depicted in Figure 1.

## Discussion and conclusion

This long-term, population-based study is including consecutively operated patients with RCC from a well-defined region in Sweden. Even though the number of patients in the study is limited, it consists of a complete cohort of RCC patients as very few patients were referred out of the region for operation. The limitation of the study is its retrospective design and the enrolment of patients who underwent radical nephrectomy for RCC in the period 1985 to 1994. Since then more tumours are detected incidentally and the standard of care has changed. However, the proportion of incidentally detected RCC in the present study is similar to contemporary series [18,19]. Also, the size of the data set limited the number of adjustment variables that could be included in the multivariate survival model. The results of this model are, however, largely confirmed by a simple Kaplan-Meier analysis. Also, as a sensitivity analysis we have re-run the model including pTstage (1–2 vs. 3–4). Generally, the contrasts between LNI, VI and SDM categories were not considerably different in this enlarged model (data not shown).

The strength of the study is its long-term follow-up and the high quality of follow-up data that were obtained from institutions providing health service, local GPs or population register.

The symptoms of RCC at presentation have been associated with poor outcome [3,18,20]. In univariate analysis we did find a survival advantage in favour of incidentally detected RCC compared with symptomatic RCC. However, in keeping with the findings of Ishimura et al. [20] and Gudbjartsson et al. [21], the independent prognostic significance of mode of presentation was not confirmed in multivariate analysis. In agreement with other studies, [4,22] we found that elevated ESR at presentation was associated with more aggressive disease and poorer outcome for both clear cell and papillary RCC (data not shown). Contrary to the study of Sengupta et al. [4] the frequency of elevated ESR among our patients was not significantly different between these two histological subtypes.

In general, distant metastases at operation have a profound adverse impact on survival after radical nephrectomy for RCC. However, some patients with SDM have a more indolent disease course after radical nephrectomy and are expected to survive for more than five years. In our study 10% of patients were alive after seven years of follow-up. The patients with metastases in lung or bone had a poorer prognosis than those with metastases limited to other organs. The lung was the most prevalent site of metastases in one third of the patients in whom the tumour invaded the renal veins or the vena cava. Confirming the findings of Ljungberg et al. [6] and Zisman et al [23], these studies are lending support to the notion that most metastases in such patients probably develop haematogenous rather than through the lymphatic system.

Lymph node invasion has been shown in several studies to adversely affect the survival after nephrectomy for RCC [7,23-25]. The reported incidence of LNI among patients treated with radical nephrectomy and lymph node dissection is varying from 3.3% to 14.2% [23,26] depending on the study population and the time period. In the era of modern imaging techniques, the incidence of positive lymph nodes has decreased to 3% to 5%. In the present series, 21 patients (10.7%) had lymph node metastases. The relatively high incidence of positive nodes is reflecting the patient selection as 45 patients with SDM underwent radical nephrectomy. Three quarters of the node positive patients had synchronous distant metastases, which are in line with the findings of an autopsy study [27]. Patients harbouring lymph node metastases had a sinister prognosis and in these patients the presence or absence of SDM and VI did not influence the outcome. However, in lymph node negative patients we were able to define subgroups with significant different survival according to presence and absence of SDM or VI or both. Patients with tumour invasion of the vena cava or the renal veins as the only adverse feature had a significantly longer survival than those with only distant metastases. Nesbitt et al. [24] and Tsuji et al. [25] reported that in patients operated for RCC with thrombus in the vena cava, distant metastases at the time of operation did not significantly decrease the survival for those who were node negative whereas patients with lymph node metastases had a significant shorter survival. Ljungberg et al. [6] performed preoperative staging and no extensive lymph node dissection and reported a significantly shorter CSS of patients with SDM and VI compared with those with SDM only. However, it remains unclear whether the status of the lymph nodes was included in the analysis.

Only few studies have analysed survival by comparing N1/N2M1 and N0M1 disease; most patients with M1 disease are grouped together regardless of lymph node status. In the multivariate analysis of interactions of SMD, VI and LNI on survival, LNI showed a significant impact on survival only for the patients in whom we found no distant metastases or venous invasion. In this subgroup of patients there was a hazard ratio of 9.0 for later death of RCC. This finding implies that once RCC has spread to the lymphatic system the risk of haematogenous spread to other regions is high, and it is likely that few patients would benefit from an extensive lymph node dissection. In accordance with other reports, the impact of VI on survival was highest for patients free from nodal and distant metastases, and was insignificant in patients with both LNI and SDM [7,28].

Our findings underline the prognostic importance of the status of the lymph nodes. LNI, SDM, ESR, and VI were independently associated with CSS after radical nephrectomy. LNI provided the strongest prognostic information for patients without SDM or VI whereas SDM and VI had strongest impact on survival when there was no nodal involvement. These findings imply that once RCC has spread to the lymphatic system the risk of haematogenous spread to other regions is high and it is likely that few patients would benefit from extensive lymph node dissection.

## Competing Interests

The authors declare that they have no competing interests.

## Authors' contributions

DPZ conceived the study, carried out acquisition of data, participated in analysis and wrote the manuscript. LB and TS carried out the pathohistological analysis of tumour specimens and have been involved in drafting and revising the manuscript. TWL performed the statistical analysis and interpretation of data and contributed to the study design. SAH participated in the design of the study, have been involved in drafting and revising the manuscript critically for important intellectual content. All authors read and approved the final manuscript.

## Pre-publication history

The pre-publication history for this paper can be accessed here:


